# Surface processes during purification of InP quantum dots

**DOI:** 10.3762/bjnano.5.135

**Published:** 2014-08-06

**Authors:** Natalia Mordvinova, Pavel Emelin, Alexander Vinokurov, Sergey Dorofeev, Artem Abakumov, Tatiana Kuznetsova

**Affiliations:** 1 Department of Chemistry, M.V. Lomonosov Moscow State University, 119991, Moscow, Russian Federation; 2 Electron Microscopy for Materials Research (EMAT), University of Antwerp, Groenenborgerlaan 171, B-2020 Antwerp, Belgium

**Keywords:** cadmium-free material, electrophoresis, luminescence, precipitation, purification, quantum dots, semiconductors

## Abstract

Recently, a new simple and fast method for the synthesis of InP quantum dots by using phosphine as phosphorous precursor and myristic acid as surface stabilizer was reported. Purification after synthesis is necessary to obtain samples with good optical properties. Two methods of purification were compared and the surface processes which occur during purification were studied. Traditional precipitation with acetone is accompanied by a small increase in photoluminescence. It occurs that during the purification the hydrolysis of the indium precursor takes place, which leads to a better surface passivation. The electrophoretic purification technique does not increase luminescence efficiency but yields very pure quantum dots in only a few minutes. Additionally, the formation of In(OH)_3_ during the low temperature synthesis was explained. Purification of quantum dots is a very significant part of postsynthetical treatment that determines the properties of the material. But this subject is not sufficiently discussed in the literature. The paper is devoted to the processes that occur at the surface of quantum dots during purification. A new method of purification, electrophoresis, is investigated and described in particular.

## Introduction

Colloidal semiconductor nanocrystals (NCs) have been studied extensively for the last two decades due to their unique size-dependent optical properties and their potential applications in the areas of photoluminescent devices, light-emitting diodes, displays, photodetectors, photovoltaic devices, solar cells and biological imaging [1–2]. III–V Nanocrystals are of increasing interest as a replacement for toxic CdSe quantum dots (QDs). Among them, InP QDs attracted the most attention because they are not toxic and have a broad photoluminescence color range in the visible spectrum. There are several methods to synthesize InP NCs. The most popular synthetic route is the reaction of an indium salt with tris(trimethylsilyl)phosphine (P(TMS)_3_) in a solvent with a high boiling point at high temperatures [3–4]. This phosphorous precursor has a number of disadvantages and should be replaced with another one, for example PH_3_ [5–6].

Right after synthesis, QDs should be purified from byproducts. There are a lot of strategies for the size and shape-selective purification of nanoparticles [7]. Size-selective precipitation is one of the most important separation technique used widely in colloid chemistry. It relies on the fractional precipitation from a “good” solvent by addition of a “bad” one [8]. Another simple and effective method widely used in biological and biochemical research, protein chemistry and pharmacology is electrophoresis [9]. Electrophoretic techniques can separate charged objects in a uniform electric field, but this technique is usually applied to aqueous solutions of QDs [10].

In this paper we compare two methods of InP QDs purification: traditional precipitation and a new electrophoretic technique in an organic solvent and describe the surface processes that occur during purification.

## Experimental

### Materials and equipment

For synthesis we used high-purity argon, PH_3_ (high purity, mixture with argon 1:1), anhydrous indium acetate (In(OAc)_3_, Aldrich, 99.9%), myristic acid (98%, Fluka). Toluene, acetone and octadecene (ODE, 90%) were used as solvents.

Absorption spectra were measured at room temperature with a Varian Cary 50 spectrophotometer in a 1 cm quartz cuvette. Photoluminescence (PL) spectra were measured at room temperature with an Ocean Optics 4000 USB spectrometer calibrated by using a 2600 K W-lamp. Excitation of PL was carried out by using a 405 nm continuous laser LED (40 mW). Powder X-ray diffraction (XRD) patterns were taken on a Rigaku D/MAX 2500 diffractometer using Cu Kα radiation (λ = 1.540598 Å). Transmission electron microscopy (TEM) was performed on a LEO912 AB OMEGA microscope. High-angle annular dark field scanning transmission electron microscopy (HAADF-STEM) imaging and energy-dispersive X-ray (EDX) analysis have been performed with an aberration-corrected FEI Titan 80-300 “cubed” microscope equipped with a Super-X detector and operated at 200 kV. The TEM specimens were prepared by placing drops of the QD suspension onto a holey carbon grid. IR spectra were taken on a Perkin-Elmer “Frontier” FTIR-spectrometer.

### QDs synthesis and purification

Synthesis of InP NCs was performed as described in [5]. Myristic acid was used as a stabilizer. The mixture of precursors (0.1 mmol of In(OAc)_3_ and 0.3 mmol of myristic acid in 2 mL of ODE) was heated to 260 °C in neutral Ar atmosphere under stirring. After the complete dissolution of precursors about 3 mmol PH_3_ was bubbled through the solution. The mixture was maintained at the reaction temperature for 15 min, then rapidly cooled and purified.

To purify the synthesized NCs, we carried out the precipitation with acetone or an electrophoretic technique. NCs precipitated with acetone were separated by centrifugation and re-dissolved in toluene. Electrophoresis was carried out in acetone in an U-shaped quartz tube, the distance between two electrodes is 10 cm. The QDs were placed near the cathode and deposited on the anode made of stainless steel at the voltage of 1 kV and were also re-dissolved in toluene. Purification was performed repeatedly and IR spectra were taken each time, and 30 days after the last purification. TEM was also performed after the final purification of QDs. The reaction mixture and the last fraction of precipitate with acetone were vacuum-sealed in two ampoules to monitor changes in the intensity of luminescence.

## Results and Discussion

**Figure 1:**
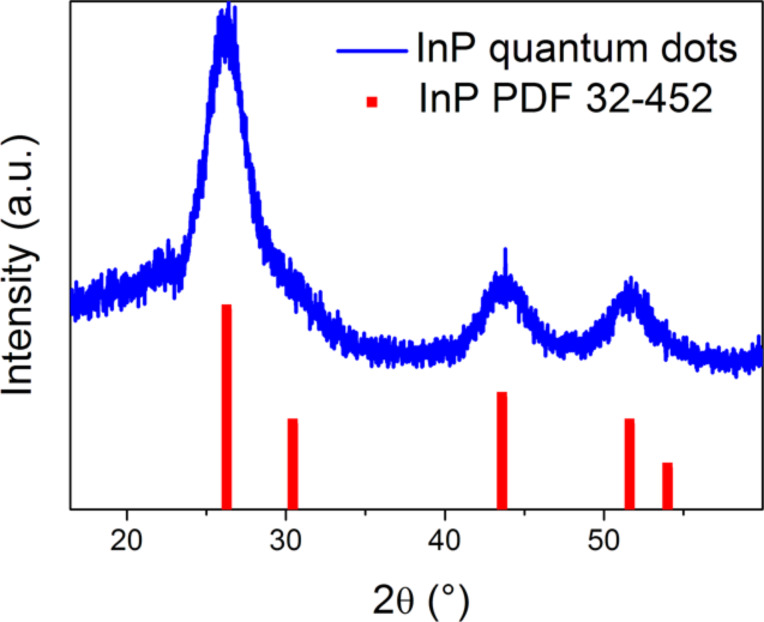
X-ray powder diffraction pattern of the synthesized InP QDs.

**Figure 2:**
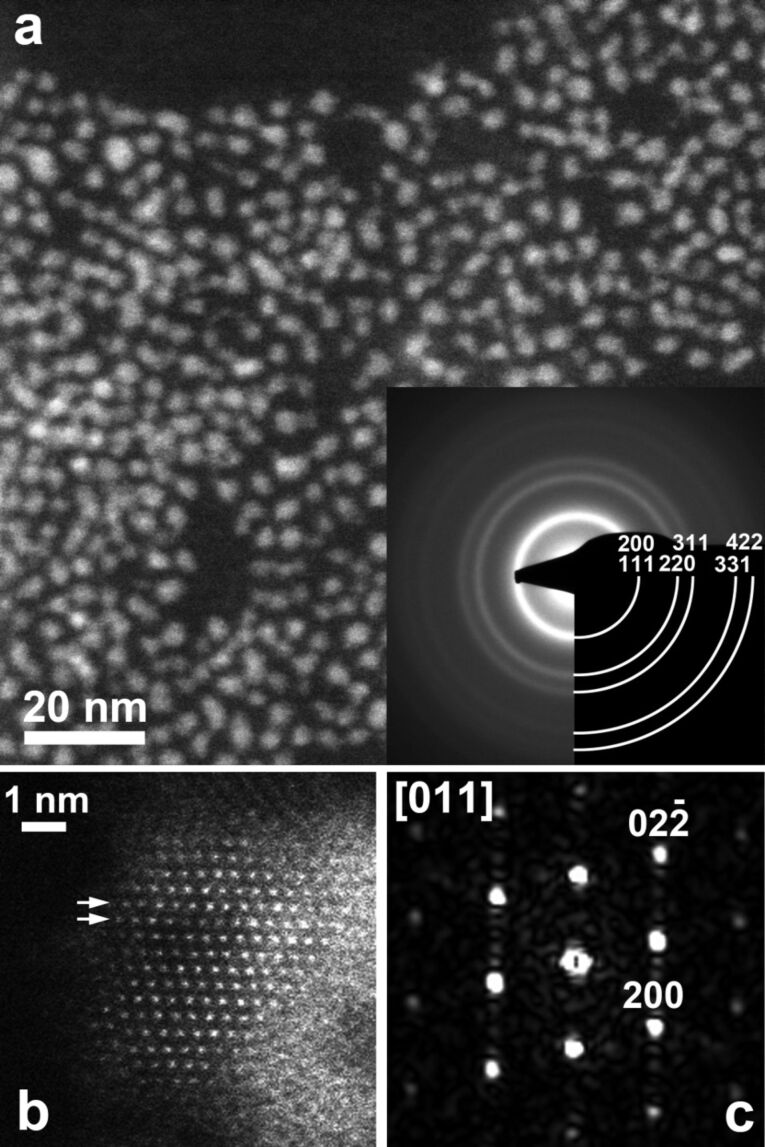
(a) Overview HAADF-STEM image of InP QDs. The ring electron diffraction pattern (insert) is indexed on a face-centered cubic lattice with *a* ≈ 5.9 Å. (b) High resolution HAADF-STEM image of the [011]-oriented QD. Planar defects (stacking faults) associated with {111} close-packed planes are marked with arrows. (c) Fourier transform of the HAADF-STEM image in Figure 2b indexed with a face-centered cubic InP unit cell. Weak extra spots along the 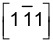 reciprocal lattice direction are because of stacking faults.

**Figure 3:**
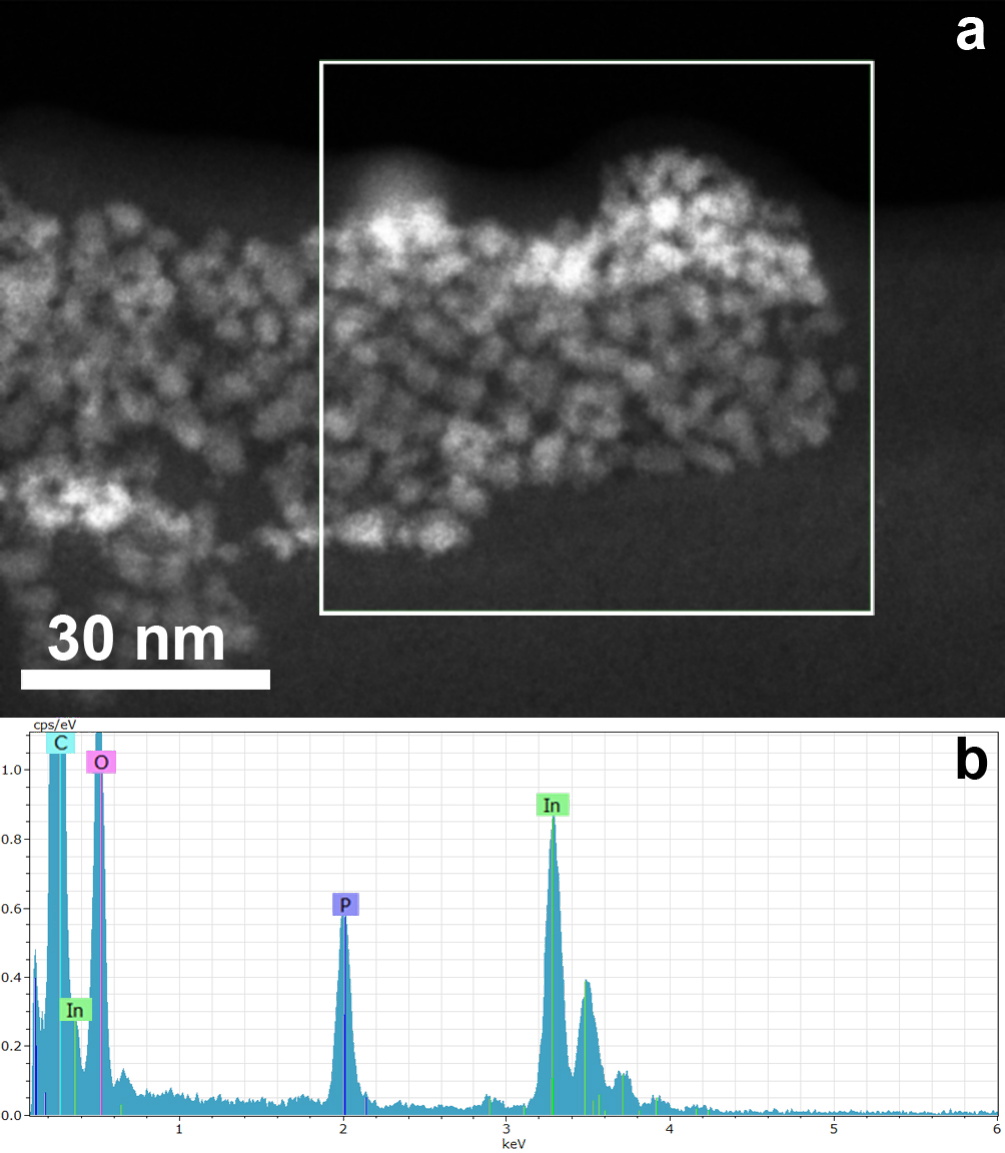
HAADF-STEM image of InP QDs with the area selected for the EDX analysis (a) and the EDX spectrum (b).

XRD shows that the QDs are pure InP nanocrystals (Figure 1). Figure 2a shows an overview HAADF-STEM image of the QDs that have a size ranging between 2 and 7 nm. The ring electron diffraction pattern (insert in Figure 2a) shows that the QDs are crystalline with the face-centered cubic InP crystal structure (*a* ≈ 5.9 Å). The HAADF-STEM image of a [011]-oriented QD and its Fourier transform in Figure 2b and Figure 2c, respectively, confirm that the QDs have the face-centered cubic InP crystal structure and demonstrate occasional stacking faults related to a replacement of “cubic” layers with “hexagonal” layers. The EDX analysis confirms the presence of both In and P in QDs (Figure 3) and reveals the In:P ratio of 1.14(2):0.86(2). The EDX spectra reveal that the QDs are noticeably oxidized.

**Figure 4:**
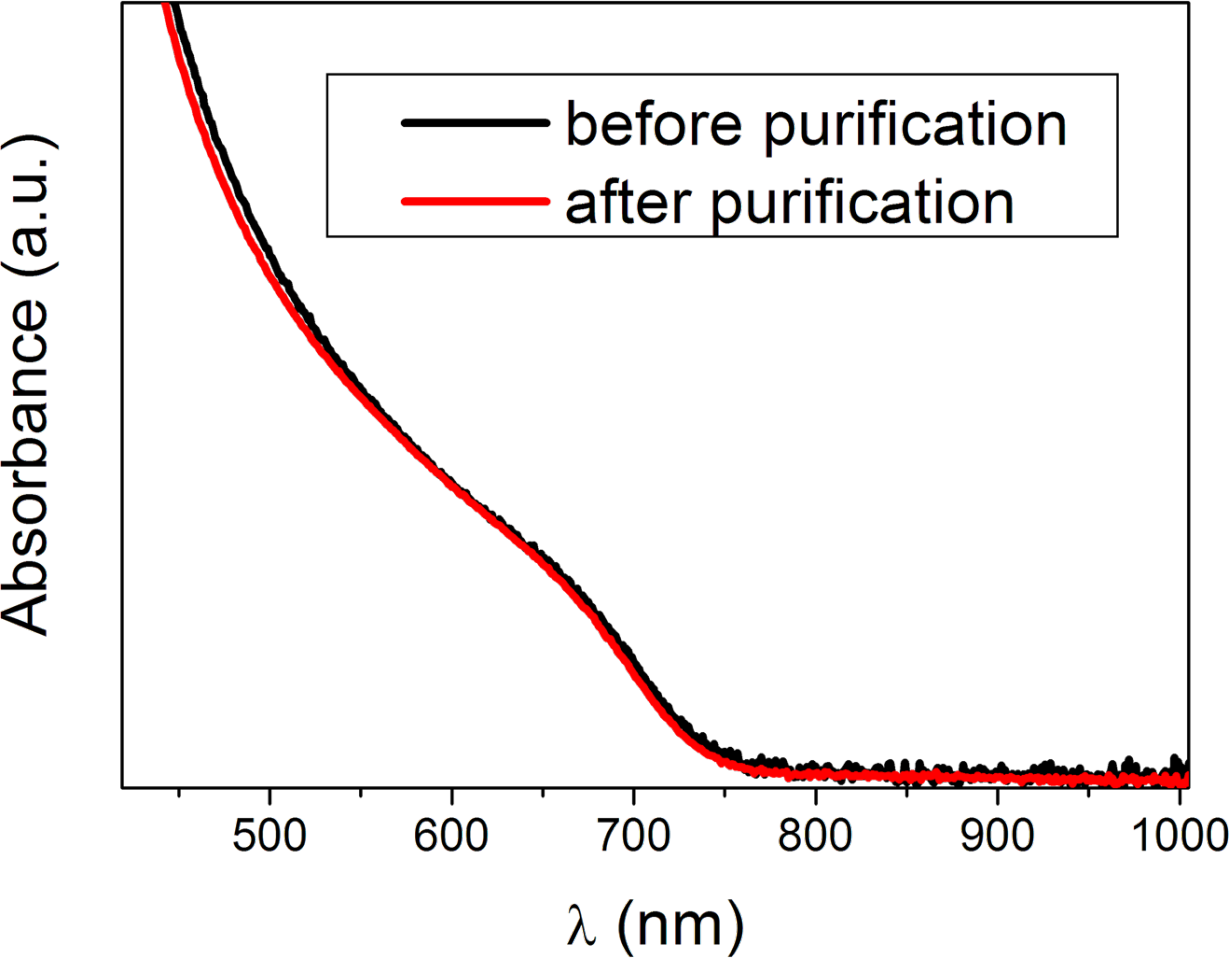
UV–vis absorption spectra of InP QDs before purification and after the last precipitation with acetone.

**Figure 5:**
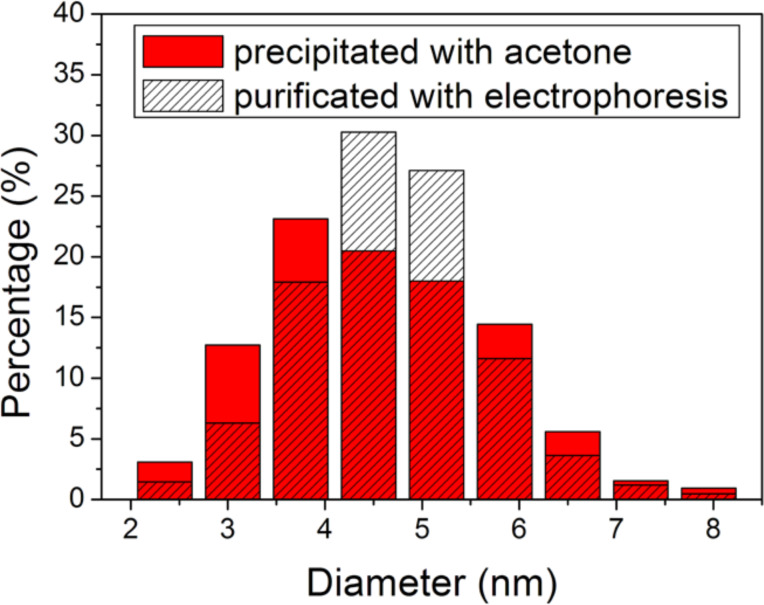
Size distribution of purified InP QDs.

Figure 4 shows the UV–vis absorption spectra of InP QDs before purification and after the last precipitation with acetone. These spectra are the same. The spectrum of the sample purified with electrophoresis completely matches with these spectra. Thus, we can conclude that small as well as large particles could be completely precipitated and the size distribution of NCs is the same for different types of purification. The excitonic peak is diffuse. This indicates that QDs are polydisperse. The size distribution obtained from TEM is shown in Figure 5.

### Precipitation with acetone

**Figure 6:**
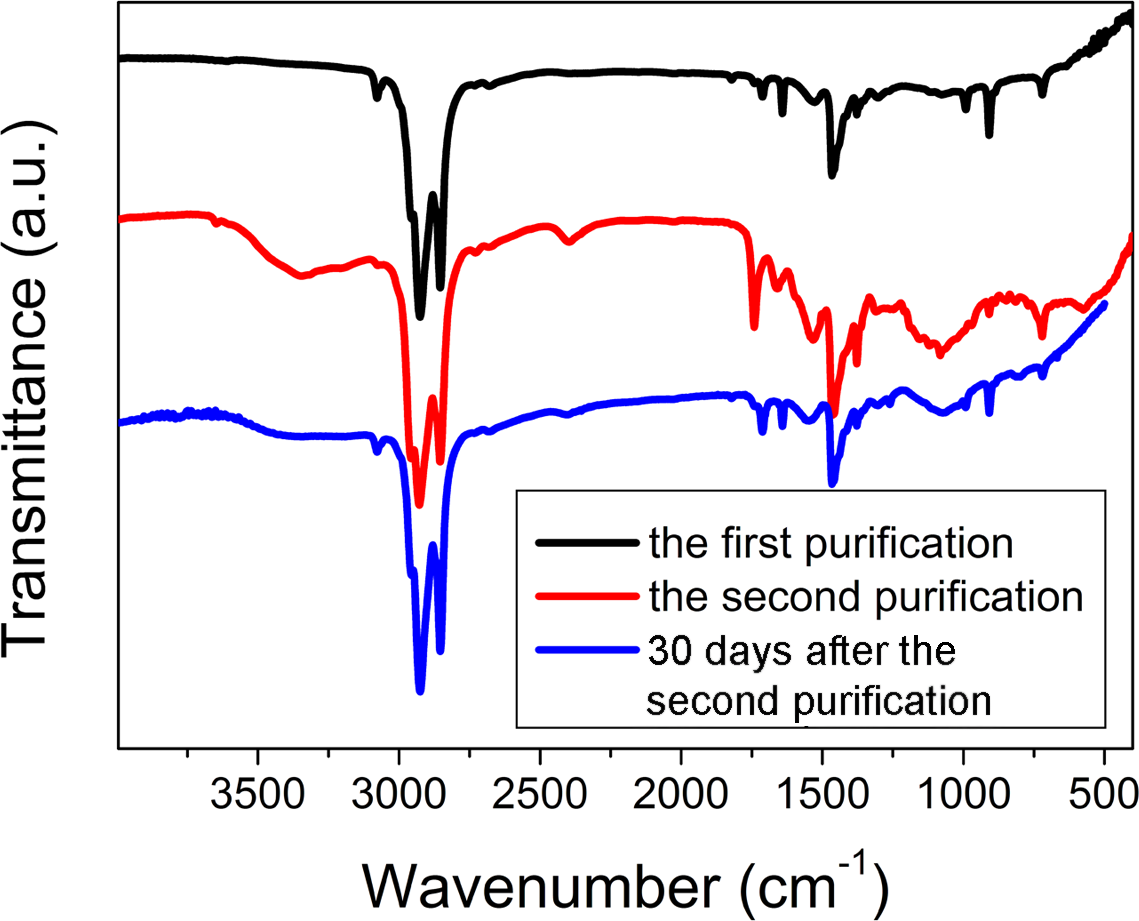
IR spectra of QDs precipitated with acetone.

Figure 6 shows how the IR spectra changes during the precipitation with acetone and after 30 days from the last purification. The IR spectra of the second and the following steps of purification were identical. Therefore, we consider only the second step of precipitation. The assignment of the vibrations of the samples is given in Table 1.

**Table 1:** Assignment of the vibrations of the samples.

wavenumber [cm^−1^]^a^	assignments	comments

3600–3000	O–H st	myristic acid
3095–3075	=CH_2_ st	ODE
3000–2840	C–H st	
2440–2275	P–H st	PH_3_
1765–1645	С=О st	myristic acid
1690–1635	C=C st	ODE
1610–1550	(COO^−^) st as	
1470–1430	CH_3_ δ as or CH_2_ δ	
1450–1400	(COO^−^) st sy	
1300–800	–OH	myristic acid
1005–985	CH=CH_2_	ODE
920–900		
770–720	–(CH_2_)_n_–	

^a^[11–12].

**Figure 7:**
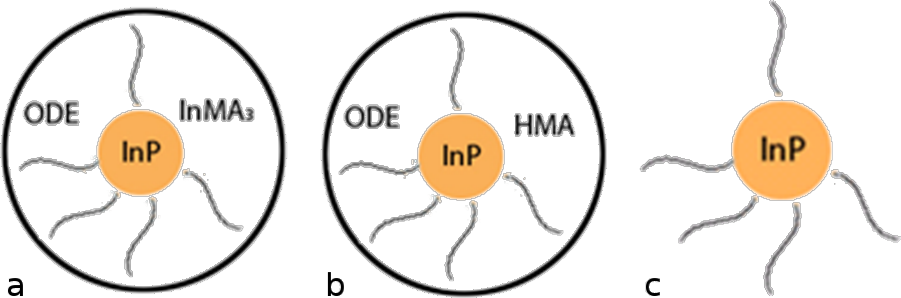
Scheme of QD at different steps of purification. (a) after the first purification, (b) after the second precipitation with acetone, (c) after the second purification with electrophoresis.

Apparently, the sample after the first precipitation contained a large amount of ODE. Besides, this IR-spectrum shows the presence of COO^−^ groups in the sample and the absence of C=O and O–H groups. Thus, there was only indium myristate (In(MA)_3_) and no myristic acid (HMA) in the sample. After the second purification peaks, that we have assigned to HMA, appear. Before purification there was some excess of unreacted In(MA)_3_ in the sample that was almost removed during the first purification, but a small part of In(MA)_3_ was still dissolved in ODE that covered the surface of the QDs. So, we suppose that after the first precipitation with acetone the QDs are nanocrystals of InP stabilized with the myristate anion and covered with an ODE layer that contains dissolved In(MA)_3_ (Figure 7a).

During the second precipitation In(MA)_3_ in the ODE layer is hydrolyzed because of the water contained in acetone according to the following reaction:


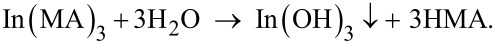


In(OH)_3_ is insoluble in ODE and leaves this layer of the QD. A QD after the second precipitation with acetone is shown in Figure 7b. Myristic acid is formed by the hydrolysis and most probably dissolves the surface of the QD under the formation of PH_3_ according to the next reaction:


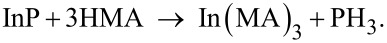


This hypothesis is confirmed by the IR spectrum of the sample precipitated for the second time, which contains a peak that corresponds to P–H vibrations. After 30 days the quantity of free myristic acid decreases (Figure 6) and the quantum yield (QY) of luminescence increases (reaches more than 1%) as the result of the stabilization on the QDs surface.

It is known that the efficiency of QD luminescence increases a few days after synthesis because of the oxidation of the QDs surface [4]. In order to show that the increasing luminescence intensity is indeed a result of hydrolysis during precipitation, two aliquots (reaction mixture and precipitated samples) were vacuum-sealed. The QY of the reaction mixture did not increase. But the QY of the precipitated QDs increased and reached more than 1% after 30 days.

### Electrophoretic technique

**Figure 8:**
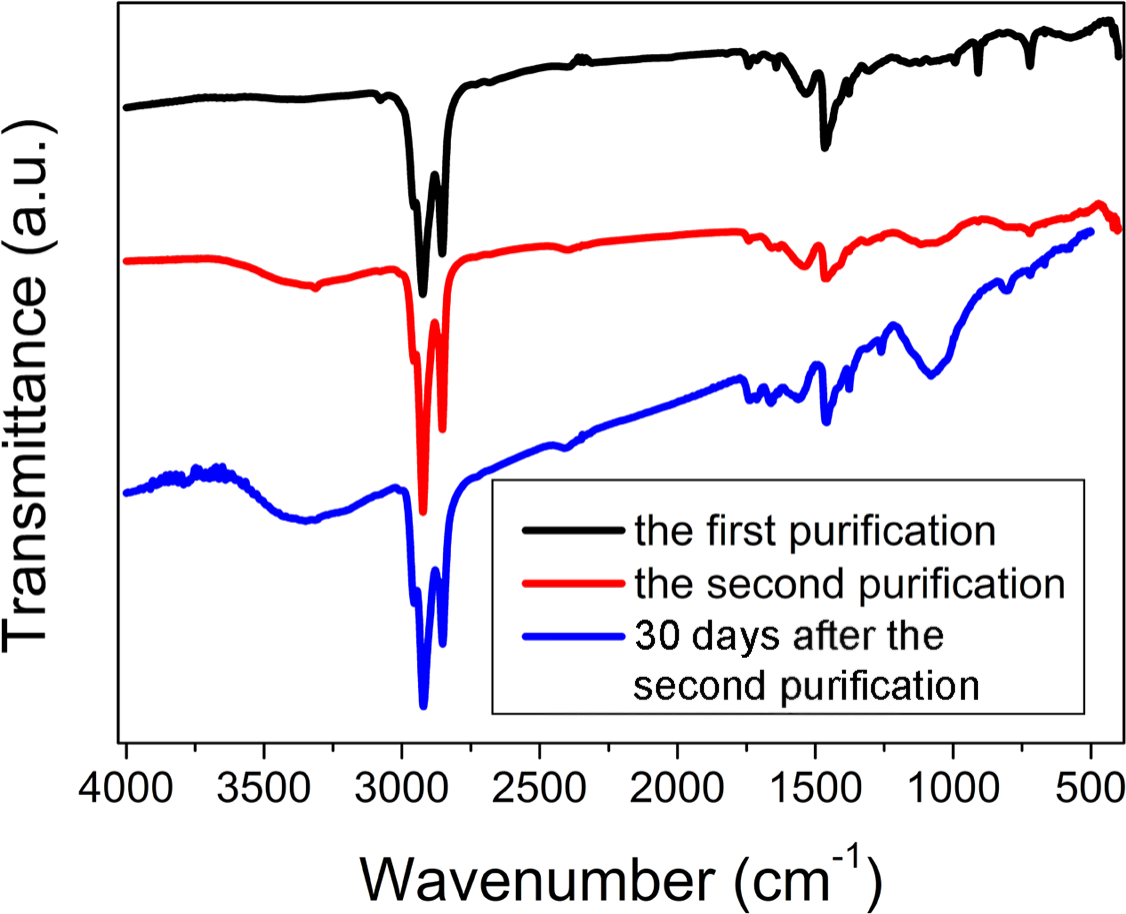
IR spectra of QDs purified with the electrophoresis.

The electrophoretic technique enables us to purify QDs from ODE that is dissolved in the stabilizing shell. After the first electrophoretic purification small peaks corresponding to ODE are noticeable in the IR spectrum and after the second purification there are no ODE peaks in the IR spectrum of the sample (Figure 8). Similar to the precipitation with acetone, we consider only the second step of purification with electrophoresis because the properties of the samples obtained after the second purification are identical. During the first purification excess ODE and In(MA)_3_ are removed from the QDs surface as it happens during the first precipitation with acetone. During the second purification ODE completely leaves the QDs surface and the QDs look as shown in Figure 7c. After 30 days MA^−^ that stabilizes the QD is hydrolyzed because of traces of water in air (Figure 8). Because of the poor stabilization of the QDs surface the quantum yield of luminescence does not increase and is less than 0.5%.

**Figure 9:**
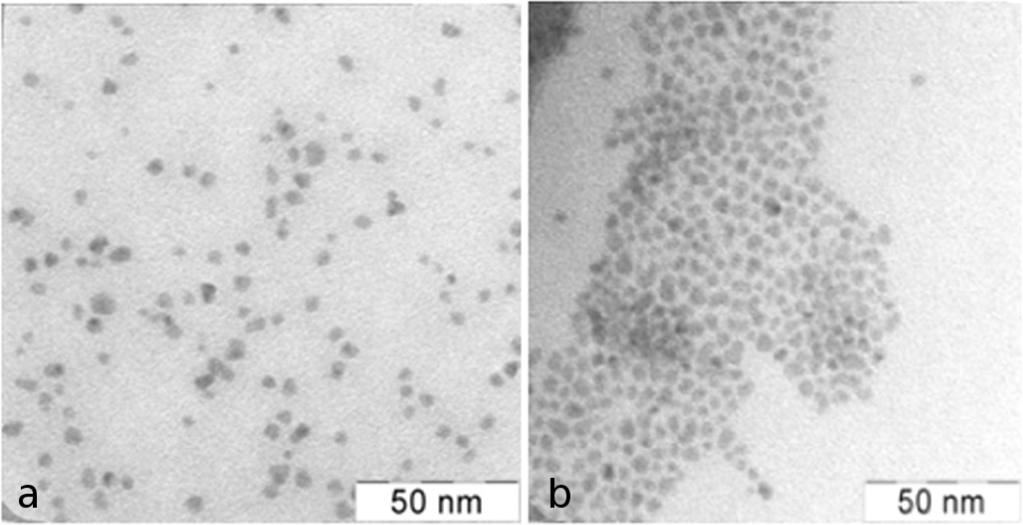
TEM images of InP QDs (a) after the second precipitation with acetone, (b) after the second purification with electrophoresis.

The comparison of the TEM images for two types of purification is shown in Figure 9. QDs purified with electrophoresis are grouped together, the distance between two QDs is about 1–3 nm (Figure 9b). QDs precipitated with acetone are distributed more randomly and are not aggregated because of the ODE layer which prevents the aggregation of particles.

### In(OH)_3_ formation

In our previous paper [5] we described a low-temperature (160–200 °C) synthesis of QDs that is accompanied by the formation of In(OH)_3_. We supposed that indium acetate, which contains traces of water, causes the formation of In(OH)_3_. The scheme of purification mentioned above explains this fact by the hydrolysis of unreacted In(MA)_3_. Actually, solutions of samples stabilized with HMA and synthesized at lower temperatures (160–200 °C) are almost transparent; the concentration of InP QDs is very low. So on the other hand, the concentration of unreacted In(MA)_3_ is very high. To confirm our suggestion, we performed one additional synthesis. During the synthesis excess PH_3_ was bubbled through the precursors solution heated to 160 °C. The solution became dark brown and very saturated. XRD showed that after purification there is only InP and no In(OH)_3_ in the sample. Our investigations of the reaction at different temperatures enables us to conclude that at lower temperatures the rate of the reaction between In(MA)_3_ and PH_3_ is very low and the hydrolysis of unreacted In(MA)_3_ during the purification leads to the formation of In(OH)_3_.

## Conclusion

We have compared two methods for the purification of QDs: precipitation with acetone and an electrophoretic technique. Electrophoresis is a fast and efficient technique that enables the purification of QDs from byproducts, including ODE, that contaminate the sample. During the precipitation with acetone ODE admixture could hardly be removed. However, electrophoresis has some limitations: After purification through electrophoresis no luminescence is observed. To increase the quantum yield of QDs purified in such a way a postsynthetic treatment, for example photoetching with HF [13], is required. Luminescence QY of QDs precipitated with acetone increases up to 1% because of the hydrolysis of excess indium myristate, which is dissolved in ODE. We have proposed the structure of QDs after each step of both types of purification and explained the differences between them.

We also explained the formation of In(OH)_3_ during the low-temperature synthesis by the hydrolysis of unreacted indium myristate during the purification. To prevent the formation of In(OH)_3_ a greater amount of PH_3_ should be bubbled through the precursors solution.
